# A Novel CNN-LSTM Algorithm for Strain Time Series Prediction of Orthotropic Steel Bridge Decks

**DOI:** 10.3390/s26144399

**Published:** 2026-07-10

**Authors:** Haiping Zhang, Miao Meng, Lei Zhao

**Affiliations:** School of Civil Engineering, Hunan University of Technology, Zhuzhou 412007, Chinaygxxxxz@163.com (L.Z.)

**Keywords:** OSBDs, strain time series prediction, CNN-LSTM, wavelet decomposition

## Abstract

Accurately predicting the strain time series of orthotropic steel bridge decks (OSBDs) is highly challenging due to their strong stochasticity and nonlinear characteristics. This paper proposes a hybrid prediction framework integrating wavelet decomposition with a cascaded Convolutional Neural Network and Long Short-Term Memory architecture. Initially, the raw strain signals are decoupled into temperature-dominated low-frequency trends and vehicle-induced high-frequency dynamic components using the 6-level Daubechies 10 wavelet transform. Subsequently, a deep architecture comprising three CNN layers and two LSTM layers is constructed to precisely extract and learn the local spatial features and long-term temporal dependencies of the decoupled signals. Based on real-world monitoring data, the proposed model is comparatively evaluated against baseline models, including CNN-GRU, LSTM, and Gated Recurrent Unit (GRU), across three time horizons: 24 h, 1 h, and 10 min. The results demonstrate that the proposed method consistently exhibits superior predictive performance across multiple scales. Specifically, the mean absolute percentage error (MAPE) is strictly maintained below 0.6% across all tested horizons, with an R^2^ reaching 0.961. Furthermore, the single-step inference latency is merely 0.63 milliseconds, which is significantly lower than conventional sensor acquisition intervals. This decouple-then-predict analytical framework effectively avoids the feature interference typically encountered when a single network directly processes complex mixed signals. Moreover, while strictly satisfying real-time computational constraints, it provides an undistorted, high-fidelity data foundation for future online fatigue evaluations and continuous state tracking of bridge structures.

## 1. Introduction

OSBDs, characterized by their light weight and good aerodynamic performance, are widely used in long-span bridges. However, fatigue cracks are prone to developing at the welds of existing OSBDs, potentially compromising structural integrity. Strain monitoring at welded joints provides an effective means to quantify accumulated fatigue damage in these critical structural details, while abrupt strain variations lacking timely warning mechanisms may precipitate catastrophic failure consequences [1,2]. High-fidelity strain forecasting is a fundamental prerequisite for reliable fatigue life assessment. Although this study focuses primarily on advanced strain prediction, such accuracy directly dictates the reliability of downstream fatigue analysis. Traditional models often suffer from peak clipping or signal smoothing, which distorts vehicle-induced stress amplitudes and causes exponential errors in cumulative damage calculations. By precisely reconstructing high-frequency vehicular impacts without amplitude loss, the proposed framework delivers an undistorted dynamic load spectrum, providing robust data support for future real-time fatigue evaluation. The strain at the welds of in-service OSBDs is primarily induced by vehicle loads, temperature variations, and environmental loads [3,4]. Consequently, the strain data typically exhibit stochasticity and strong nonlinear characteristics [5,6]. Furthermore, commercial off-the-shelf strain sensors exhibit a typical service lifespan of 3 to 5 years, falling significantly short of the bridge’s 100-year design life. This discrepancy necessitates 6 to 8 sensor replacements over the structure’s entire service life, resulting in a substantial increase in lifecycle operational expenditures for structural health monitoring systems [7,8,9,10]. Strain monitoring serves as a critical indicator for bridge serviceability assessment. However, raw monitoring signals exhibit multi-source coupled characteristics requiring decoupling analysis. Conventional time-series models (ARIMA, SVM) are typically inadequate in capturing high-dimensional nonlinear relationships, while single deep learning architectures face feature extraction bottlenecks that fail to reconcile the modeling conflict between localized feature resolution and long-term dependency capture [11,12,13]. Therefore, the development of strain prediction methods for welds in in-service OSBDs is urgently needed.

Previous studies have shown that the strain of OSBDs primarily consists of vehicle-induced strain [14], temperature-induced strain [15], and white noise-induced strain [16]. Directly applying machine learning methods to the initially monitored strain data struggles to guarantee both prediction accuracy and efficiency; therefore, classification and separation of the monitored strain time series data are necessary. Current research focuses primarily on separating the temperature effects from strain monitoring data. Common methods include wavelet decomposition, empirical mode decomposition (EMD), and ensemble empirical mode decomposition (EEMD) [17,18,19]. Chen et al. [20] proposed a combined denoising method using EEMD and an improved wavelet threshold function, effectively improving signal processing accuracy. Xu et al. [21] significantly enhanced the learning ability for nonlinear data using wavelet decomposition and a dual-attention LSTM (DA-LSTM) network. However, these methods still have limitations when dealing with complex environmental influences. Harrou et al. [22] combined the advantages of wavelet denoising and recurrent neural networks (RNNs), successfully capturing the nonlinearity and temporal dependencies of time series data, but improvements are still needed in handling long-term trend changes.

Strain prediction methods based on CNNs offer potential for assessing structural safety even with missing sensors or data defects; however, their applicability under sparse or distorted data conditions requires further strengthening [23]. In data prediction, Abinash et al. [24] utilized LSTMs to predict time-series data, providing valuable insights for bridge strain prediction. While neural network-based prediction of bridge strain data is feasible, its dynamic adaptability still needs validation [25,26]. To address this, Zhao et al. [27] proposed a strain field analysis model based on an improved Generative Adversarial Network (GAN), effectively predicting strain distribution and identifying structural damage states through intelligent analysis, offering new approaches for numerical analysis and structural design. However, limitations in GAN model training stability and large-scale data processing remain challenges. Yao et al. [28] employed a Time GAN to enhance monitoring data, improving the ability to extract data correlations, but its high computational cost may limit practical applications. The development of minute-scale prediction models using LSTM networks addressed accuracy issues stemming from single-point input and lag effects, but performance in complex multi-point environments requires optimization [29].

Table 1 lists the comparison of strengths and weaknesses of time series prediction algorithms. Regarding the vehicle-induced strain and temperature-induced strain, several researchers have conducted studies [30,31,32,33]. For example, Luo et al. [34] proposed the computer vision technology Surface Vision, which integrates multiple algorithms to achieve high-precision strain measurement in complex field environments and develops a prediction method based on strain analysis. Kuo et al. [35] constructed a deep learning architecture that fuses GNNs and LSTMs, overcoming the limitations of traditional models and enabling effective prediction of the dynamic response of various structures. The model’s performance was significantly improved through sequence padding and compression strategies. Kromanis et al. [36] developed a thermal response prediction model using robust regression, artificial neural networks (ANNs), and support vector regression (SVR) based on bridge temperature distribution. In cases of partial missing data in bridge monitoring, multi-algorithm fusion is necessary [37]. Existing research shows that embedding one-dimensional CNNs into LSTM models can improve the prediction accuracy and capability for partially missing strain data [38,39,40]. Recent studies further demonstrate the immense potential of these hybrid CNN-RNN approaches in broader bridge SHM tasks. For example, hybrid 1D-CNN-RNN models have been successfully utilized to reconstruct missing or corrupted sensor data with high accuracy [41,42], while similar cascaded deep learning frameworks have proven highly effective in detecting structural damage in urban railway bridges using linear variable differential transformer data [43]. These advancements collectively highlight the superior feature extraction and temporal modeling capabilities of hybrid neural networks in complex structural monitoring. Furthermore, recent advancements in dynamic neural networks and CNN-based architectures have demonstrated exceptional capabilities in complex industrial fault diagnosis and structural feature extraction. For instance, hybrid deep learning via multi-source signal fusion has been effectively applied to hydraulic cylinder fault diagnosis under complex conditions [44]. Similarly, improved residual networks combined with continuous wavelet transforms have shown robust performance in precision machining equipment fault diagnosis [45]. Moreover, advanced CNN frameworks have been designed for reliable bolt key-point detection in harsh industrial magnetic separator systems [46]. Inspired by these state-of-the-art CNN applications in complex operational environments, this study leverages the powerful local feature extraction capabilities of CNNs for bridge strain analysis. Wang et al. [47] utilized CNNs and multi-layer dilated LSTMs to develop a short-term load prediction model, achieving higher prediction accuracy through historical data analysis. Chen et al. [48] proposed a CNN-GRU model combined with a Bayesian optimization (BO) algorithm for hyper parameter design, demonstrating excellent generalization ability in time series prediction and accurately characterizing the nonlinear relationships between data. In the broader context of Structural Health Monitoring (SHM), continuous long-term monitoring data is inevitably obscured by environmental and operational variations (EOV), making data normalization a critical prerequisite for reliable condition assessment. For instance, recent studies, such as the continuous monitoring of a steel truss railway bridge in Italy [49], emphasize the essential role of robust data normalization strategies to filter out environmental thermal effects and isolate true structural responses. Building upon this vital SHM concept, the physical-guided decouple-then-predict framework proposed in this paper organically integrates physical-prior signal decoupling (wavelet transform) with the deep CNN-LSTM architecture. This innovative approach not only achieves high-fidelity normalization of environmental temperature drifts but also maximizes the extraction of vehicle-induced transient impacts within a unified model, thereby offering a highly integrated and robust analytical tool for modern bridge SHM systems. While existing research has made significant progress in strain monitoring and prediction, current hybrid models (e.g., VMD-LSTM) predominantly focus on mathematical signal decomposition properties while neglecting the physical generation mechanisms of vehicle-induced and thermal strains. This oversight results in non-targeted feature extraction, necessitating further improvements. Harrou F et al. [50] proposed integrated attention mechanisms and wavelet-enhanced deep learning models that have successfully captured complex spatiotemporal features in environmental and traffic systems. Their work exemplifies how hybrid deep learning frameworks have recently emerged as the mainstream paradigm for forecasting complex time series. Furthermore, recent advancements in handling strongly chaotic systems further highlight the critical necessity of rigorous signal decomposition and algorithmic stability in highly dynamic environments [51,52]. Despite these significant advancements, existing methods exhibit major limitations when applied to orthotropic steel bridge deck strain prediction. The operating strain in OSBDs is inherently a deeply coupled mixture of extremely low-frequency environmental thermal drifts and high-frequency vehicular transient peaks. Most existing studies merely deploy architectures like CNN-LSTM as generic mapping tools by feeding raw data directly into the networks. Forcing a purely data-driven network to simultaneously fit these physically conflicting characteristics from a single sequence causes severe feature confusion, ultimately bottlenecking predictive performance. Moreover, existing studies generally lack rigorous multi-scale statistical validation, making it difficult to mathematically prove the significance and stability of their performance improvements.

The motivation of this study addresses a critical bottleneck in OSBD monitoring: directly feeding mixed strain signals—where slow temperature drifts and sharp vehicle impacts are deeply entangled—into traditional networks inherently causes severe ‘feature confusion.’ To overcome this, we shift from a purely data-driven approach to a physics-guided ‘decouple-then-predict’ paradigm. By explicitly separating these conflicting signals before modeling, we provide a clean and targeted foundation for the neural network, thereby significantly enhancing its learning capability.

**Figure 1:**
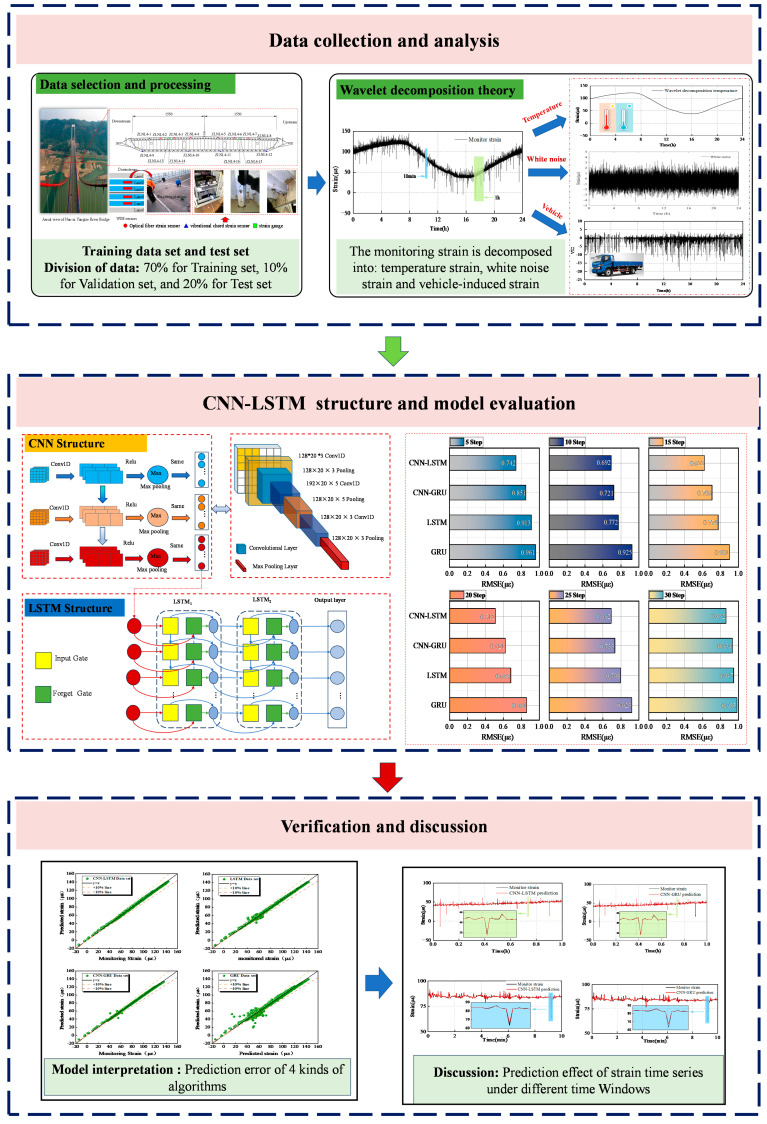
The flowchart of the strain time series prediction method.

In light of these challenges, rather than proposing a fundamentally novel neural network architecture, this paper develops a physics-guided decouple-then-predict framework for bridge strain prediction. The core contribution of this study lies in utilizing structural physical priors to govern the wavelet decomposition of highly coupled strain data, completely isolating thermal baseline drifts from transient vehicular impacts before performing deep sequence modeling using a cascaded CNN-LSTM architecture. This paper employs a 6-level Daubechies 10 wavelet transform, leveraging its suitability for processing continuously varying signals, to effectively separate the low-frequency baseline influenced by ambient temperature from the random high-frequency transient response caused by vehicle load. The model design incorporates a deep neural network architecture consisting of three CNN layers and two LSTM layers. The ability to extract local features is optimized by adjusting key parameters such as the number of filters (128→192→128) and kernel size (3→5→3), effectively capturing spatial patterns in temperature monitoring data and vehicle load data. Subsequently, the LSTM section, after adjusting the key number of units (50→100), enhances the model’s ability to capture long-term dependencies in the time series. In terms of model hyper parameter optimization, the optimal parameter combinations for each layer of the CNN and LSTM are determined, significantly improving model performance and generalization ability. Ultimately, this method effectively combines the input characteristics of multi-source data to achieve efficient prediction of bridge strain data, exhibiting higher accuracy and robustness compared to traditional methods. The flowchart of this study was showed in Figure 1.

It is important to emphasize the unique structural logic of the proposed CNN-LSTM framework. While standard CNN-LSTM architectures are common in time-series forecasting, our unique contribution lies in an explicitly physics-guided “decouple-then-predict” mechanism customized for OSBDs. Rather than relying on the network to implicitly disentangle deeply coupled physical phenomena, the framework utilizes a pre-calibrated wavelet transform to isolate structural responses. The essential characteristics of our cascaded network are thus intrinsically mapped to physical behaviors: the 1D-CNN layers are specifically configured to capture the sharp, localized gradients of transient vehicle loads, while the subsequent LSTM units are parameterized to track the long-term, slow-varying dependencies of environmental thermal drifts. This physically informed structural alignment maximizes the predictive fidelity of the conventional deep learning components.

## 2. Strain Time Series Data Prediction for OSBDs Using CNN-LSTM

### 2.1. Wavelet Decomposition Model

Strain monitoring data from OSBDs are influenced by environmental temperature, vehicle loads, white noise, and other factors (such as pedestrian loads and wind loads). The effect of vehicle loads on bridge strain is dominant, while the strain amplitude caused by other factors is relatively small. Strain caused by traffic volume is mainly concentrated in the high-frequency portion, while strain caused by temperature effects and white noise variations manifests in the low-frequency portion. These two signal components can be effectively separated using time-frequency analysis [53]. Further research reveals that temperature-induced strain typically exhibits low-frequency diurnal fluctuations, while vehicle load-induced strain is characterized by higher-frequency, transient fluctuations. In contrast, deflection changes caused by test noise are significantly smaller. Based on this understanding, the temperature component in the strain data can be effectively eliminated using wavelet packet decomposition.

To ensure that the extracted features possess structural engineering significance, this study adopts a physics-guided cascaded approach. The total measured strain in an in-service orthotropic steel bridge deck is inherently a superposition of two distinct physical phenomena: long-term, continuous environmental temperature drifts and highly transient, sharp, vehicle-induced elastic stress cycles. Directly feeding this highly coupled mixed signal into a neural network typically leads to feature confusion. Therefore, the fundamental physical and mechanical laws of the structure are utilized as prior constraints to govern the entire signal decoupling phase. Within this preprocessing paradigm, the core objective of applying the wavelet transform is to precisely map the mathematical properties of the algorithm to the afore mentioned physical behaviors. Consequently, the appropriate selection of the wavelet basis function and the decomposition level becomes a crucial step [54,55].

**Figure 2:**
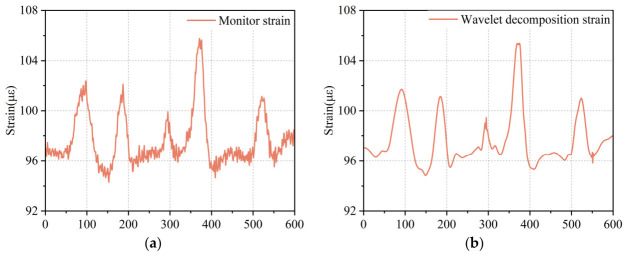
Wavelet decomposition and denoising of OSBD weld strain monitoring data. (**a**) Original strain time series; (**b**) The Wavelet decomposition of time series.

First, regarding the selection of the wavelet basis, the db10 wavelet was specifically chosen over lower-order wavelets due to its higher number of vanishing moments. Temperature-induced strain represents a macroscopic thermodynamic phenomenon, producing highly smooth and continuous polynomial curves. Although lower-order wavelets compute rapidly, their sharper, localized mathematical support struggles to reconstruct this inherent thermodynamic smoothness, often introducing artificial jaggedness or localized oscillations. Conversely, the db10 wavelet provides an optimal resolution: its higher vanishing moments ensure a rigorously smooth approximation of the low-frequency temperature trend, while its compact support effectively captures the abrupt local gradients of vehicle axle loads. As illustrated in Figure 2, the chosen db10 wavelet demonstrates excellent capability in maintaining a pristine, smooth baseline while preserving the full amplitude of the transient vehicle impacts without distortion. Second, the decomposition level was strictly calibrated to determine the precise frequency cutoff boundary. Employing a shallow decomposition maintains an excessively high cutoff frequency, causing the extracted low-frequency approximation to remain contaminated by the dynamic operational responses of slow-moving heavy trucks. Conversely, applying an excessive number of decomposition levels results in over-smoothing, where natural daily thermal variations are incorrectly filtered out and leaked into the high-frequency vehicular bands. Therefore, the decomposition depth is optimized to explicitly isolate the physical frequency gap between environmental changes and traffic loads. Given the effective sampling frequency of fs = 1 Hz established during the preprocessing phase, a 6-level decomposition strictly defines the mathematical cutoff frequency at fc = fs/26 + 1 ≈ 0.0078 Hz. This specific boundary was calibrated based on two explicit physical criteria: maximizing the Pearson correlation with the macroscopic diurnal thermal trend, and ensuring a strict zero-mean fluctuation for the isolated high-frequency vehicular elastic responses.

**Figure 3:**
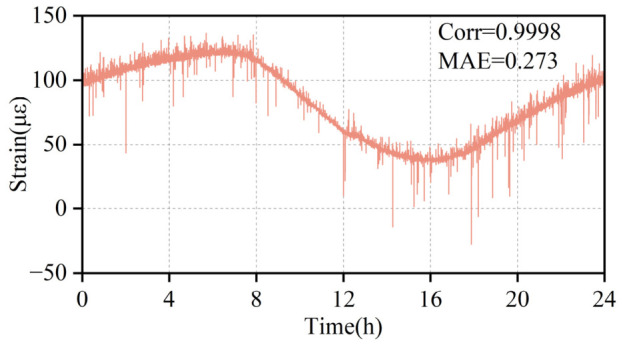
The 24-h strain history curve of the ZLNL4-1 strain sensor.

To macroscopically validate the effectiveness of this physics-guided separation method, a continuous 24-h strain signal was decomposed and evaluated against explicit structural mechanics criteria, as shown in Figure 3. Specifically, this validation logic is fundamentally predicated on two physical principles: macroscopic thermal correlation and zero-mean elastic response. Regarding the macroscopic thermal correlation, the extracted low-frequency approximation precisely mirrors the actual diurnal temperature variation in the steel bridge deck. This alignment is mathematically confirmed by an exceptionally high Pearson correlation coefficient Corr=0.9998 coupled with a remarkably low MAE=0.273, proving absolute consistency in both the macroscopic thermal trend and magnitude. Concurrently, based on the principle of local elastic recovery, transient vehicle loads generate purely elastic deformations; that is, once the load passes, the structure rapidly returns to its initial equilibrium state. This causes the isolated high-frequency vehicle detail component to strictly fluctuate around a zero-mean baseline. This zero-mean characteristic serves as a definitive physical criterion, fully confirming that the extracted high-frequency band is entirely devoid of thermal drift interference and purely represents the elastic transient stress cycles essential for fatigue analysis. By integrating these physical priors to rigorously define and validate the decoupling boundaries, the proposed framework effectively prevents the leakage of crucial fatigue-related stress range data into the temperature component, thereby achieving a pragmatic and mathematically highly robust balance in structural feature extraction.

Wavelet decomposition can be expressed as [56]:

(1) $$\psi \left(t\right)=\sum_{k=0}^{N}h\left(k\right)\phi \left(2t-k\right)$$

where h(k) are the wavelet filter coefficients, ϕ(t) is the scaling function, and ψ(t) is the mother wavelet function.

During wavelet decomposition, the first-level decomposition formula can be expressed as:

(2) $$c_{1,k=}\sum_{n}x\left(n\right)\cdot \phi_{1,k}\left(n\right)$$

(3) $$d_{1,k=}\sum_{n}x\left(n\right)\cdot \psi_{1,k}\left(n\right)$$

The formula for the *j*-th level decomposition can be expressed as:

(4) $$c_{j+1,k}=\sum_{n}c_{j,n}\cdot h\left(n\right)$$

(5) $$d_{j+1,k}=\sum_{n}c_{j,n}\cdot g\left(n\right)$$

where *g*(*n*) is the high-pass filter, the reconstruction formula can be expressed as:

(6) $$x\left(n\right)=\sum_{k}c_{j,k}\cdot \phi_{j,k}\left(n\right)+\sum_{k}d_{j,k}\cdot \psi_{j,k}\left(n\right)$$

### 2.2. CNN-LSTM Modeling

**Figure 4:**
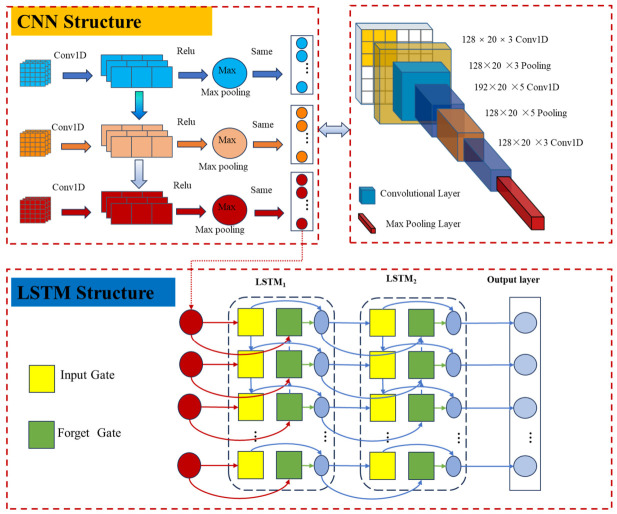
The detailed architecture of the proposed CNN-LSTM model.

This paper proposes a strain prediction model based on a CNN-LSTM, as illustrated in Figure 4. The model combines CNN and LSTM algorithms and is divided into two parts: a CNN for extracting temporal–spatial features from the strain data time series, and an LSTM responsible for load prediction. First, the CNN extracts spatial information from the strain response data at each time point through convolution and pooling operations. After feature extraction, the monitored strain response sequence is transformed into a deep feature time series containing rich local features. Subsequently, this deep feature data is input into the LSTM network, which utilizes its ability to model long-term dependencies to further achieve efficient modeling and prediction of the strain time series data. The entire method consists of two stages: data preprocessing and model training. In the data preprocessing stage, high-quality input is provided for subsequent model training through multi-source data fusion and denoising.

The first step is to normalize the monitored strain data:

(7) $$\hat{x}=\frac{x-\mu }{\sqrt{\sigma^{2}+\epsilon }}$$

(8) $$y=\gamma \hat{x}+\beta$$

where *μ* is the mean (in *μ*ε), *σ*^2^ is the variance (in *μ*$\varepsilon^{2}$), *ϵ* is a small constant dimensionless constant for numerical stability (e.g., 10^−8^), and *γ* and *β* are learnable parameters.

The second step involves convolutional feature extraction of the normalized data. The CNN consists of three parts: convolutional layers, pooling layers, and fully connected layers. Convolutional Layers: Convolutional layers slide a convolutional kernel (filter) across the input data, calculating a weighted sum of the local region. The weighted function can be expressed as:

(9) $$Y\left(i,j\right)=\sum_{m=1}^{M}\sum_{n=1}^{N}X\left(i+m-1,j+n-1\right)\cdot W\left(m,n\right)+b$$

where *X* is the input to the convolutional layer (including vehicle-related data and spatio-temporal features derived from strain), *W* is the convolutional kernel, *b* is the bias, and *Y* is the result of the convolution.

The maximum value within a local region is selected to reduce the size of the feature map, thereby reducing computational complexity and preventing overfitting.

To comprehensively quantify the complex physical characteristics of the bridge strain time series prior to network ingestion, critical signal processing metrics are incorporated into the methodology. Specifically, to mathematically evaluate the non-stationary variations and the probability distribution of the multi-source coupled strain, the signal’s skewness (*S_k_*) and kurtosis (*K_u_*) are defined as:

(10) $$S_{k}=\frac{\frac{1}{n}\sum_{i=1}^{n}{\left(x_{i}-µ\right)}^{3}}{\sigma^{3}}$$

(11) $$K_{u}=\frac{\frac{1}{n}\sum_{i=1}^{n}{\left(x_{i}-µ\right)}^{4}}{\sigma^{4}}$$

where n is the sequence length. These data characteristics objectively reflect the severity of transient vehicular impacts versus the low-frequency thermal drift baseline, explicitly guiding the subsequent convolutional feature extraction.

To properly bridge the convolutional and recurrent modules, the 3D tensor output from the final CNN layer—originally shaped as (Batch_Size, Channels, Sequence_Length) with 128 channel filters—is not globally flattened, as this would disrupt the chronological order. Instead, it undergoes a dimension transposition to (Batch_Size, Sequence_Length, 128). Through this formatting, the 128 spatial feature maps serve as the new feature dimension at each time step. The subsequent LSTM layer then processes this sequence step-by-step to capture the long-term temporal dependencies.

Step 3 focuses on the deep temporal modeling of strain time series across various prediction horizons. The core of the LSTM architecture lies in its internal memory cell, which is meticulously regulated by three sophisticated mechanisms: the forget gate, the input gate, and the output gate. In contrast to the rudimentary state updates of standard recurrent networks, these interacting gating mechanisms synergistically control the information flow by selectively discarding irrelevant historical fluctuations while assimilating vital new temporal features. This precise gating filtration not only mitigates the vanishing gradient problem but also enables the network to concurrently retain short-term transient characteristics and accurately track long-term baseline trends, thereby delivering high-fidelity predictions for continuous sequences.

The memory cell is the core part of the LSTM, used to store long-term memory. Its value is updated by weighting the input and the old state. The memory cell formula can be expressed as:

(12) $$C_{t}=f_{t}\cdot C_{t-1}+i_{t}\cdot \tilde{C_{t}}$$

where $C_{t}$ is the current memory cell, $f_{t}$ is the forget gate, $i_{t}$ is the input gate, and $\tilde{C_{t}}$ is the candidate memory cell.

The forget gate controls which information is discarded from the cell state. It is computed by applying a sigmoid activation function to the output of a fully connected layer that processes the concatenation of the current time step’s input and the previous time step’s hidden state:

(13) $$f_{t}=\sigma \left(W_{f}\cdot \left[h_{t-1},x_{t}\right]+b_{f}\right)$$

where σ is sigmoid activation function, $W_{f}$ is the weight matrix, and $b_{f}$ is the bias term.

The input gate receives *x_t_* and *h_t_*_−_ as inputs, and simultaneously combines *x_t_* and *h_t_*_−1_ with tanh to generate a new candidate state vector *C_t_*, thereby deciding which new information should be written into the memory unit *C_t_*. The formula of the input gate can be expressed as:

(14) $$i_{t}=\sigma \left(W_{i}\cdot \left[h_{t-1},x_{t}\right]+b_{i}\right)$$

(15) $$\tilde{C_{t}}=tanh\left(W_{c}\cdot \left[h_{t-1},x_{t}\right]+b_{c}\right)$$

where tanh is the hyperbolic tangent activation function, *W_i_* and *W_c_* are weight matrices, and *b*_c_ are bias terms.

At time step *t* − 1, the previous cell state *C_t_*_−1_ is added to the current input *x_t_* and the previous output *h_t_*_−1_, and then processed through the sigmoid function to select the information to be output from the memory unit. The formula for the output gate can be expressed as:

(16) $$O_{t}=\sigma \left(W_{o}\cdot \left[h_{t-1},x_{t}\right]+b_{o}\right)$$

(17) $$h_{t}=O_{t}\cdot \tanh\left(C_{t}\right)$$

where *o_t_* represents the output gate while *h_t_*_−1_ denotes the current hidden state.

**Figure 5:**
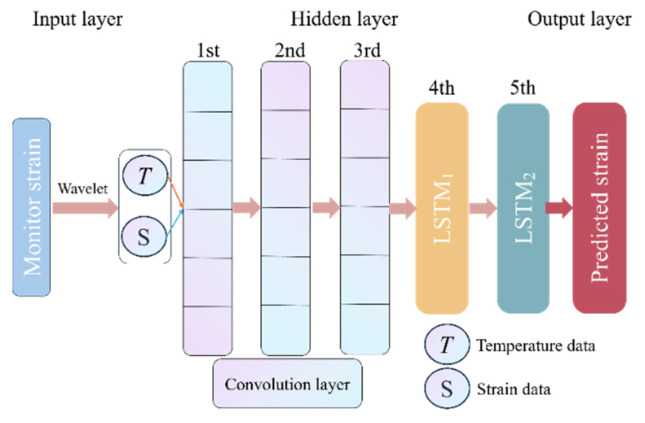
Computational Process of the CNN-LSTM Model.

Figure 5 illustrates the complete flowchart of the proposed methodology. This analytical framework operates through a systematic, end-to-end pipeline. Initially, continuous raw strain signals are acquired from an operating orthotropic steel bridge deck to establish the foundational time-series dataset. To address the highly coupled and non-stationary nature of bridge strain signals, a physical-prior intervention module utilizing the db10 wavelet transform is introduced. This explicitly decouples the complex raw signals into a low-frequency baseline driven by environmental temperature and high-frequency transient components induced by random vehicular loads. Subsequently, the cascaded CNN-LSTM architecture executes the core spatiotemporal feature extraction: a one-dimensional CNN is first employed to efficiently capture the local strain gradients induced by transient vehicles along the spatial dimension; the compressed feature sequences are then fed into an LSTM to accurately track the long-term temporal evolution trends dominated by thermal effects. Following this deep spatiotemporal feature integration, the framework outputs multi-scale strain predictions, and its predictive accuracy and computational efficiency are comprehensively evaluated.

### 2.3. Advantages of CNN-LSTM Model

We propose a CNN-LSTM model for predicting the strain time series of OSBDs due to the following reasons:

(1) **Complementarity:** The combination of CNN and LSTM leverages the strengths of both architectures. The strain time series of OSBDs are generated by multiple factors, including vehicle loading, environmental temperature, and noise, resulting in data with multiple features and high dimensionality. CNN, through feature extraction and dimensionality reduction, effectively simplifies the complexity of the time series data. The multi-layer convolutional and pooling operations enhance the model’s ability to identify patterns within the data. LSTM, with its gating mechanism, accurately captures long-term dependencies in the time series and considers the combined influence of multiple input features. This complementarity leads to improved prediction accuracy and robustness in handling complex time series data.
(2) **Enhanced Prediction Accuracy:** The CNN architecture, with its locally connected and weight-sharing design, significantly reduces the number of network parameters, decreasing model complexity and enhancing its nonlinear expressive power. This design not only helps prevent overfitting but also further improves overall prediction accuracy.
(3) **Strong Adaptability:** The vehicle-induced strain effect exhibits short-term pulse characteristics, while assessing the fatigue life of OSBDs requires accurate acquisition of long-term strain data. Therefore, strain time series prediction needs to capture both short-term variations and long-term trends. The CNN-LSTM model significantly improves the adaptability to complex data, providing comprehensive and reliable analysis and prediction capabilities for complex time series.

### 2.4. Model Evaluation Metrics

The predictive performance of the model was assessed using four common time-domain evaluation metrics: the coefficient of determination (*R*^2^), root mean squared error (RMSE), mean absolute error (MAE), and MAPE. These metrics, defined in Equations (19) through (22) respectively, provide a comprehensive evaluation of both prediction accuracy and model robustness [57,58].

(18) $$MAE=\frac{1}{m}\sum_{i=1}^{m}|y_{i}-\hat{y_{i}}|$$

(19) $$RMSE=\sqrt{\frac{1}{m}\sum_{i=1}^{m}{\left(y_{i}-\hat{y_{i}}\right)}^{2}}$$

(20) $$R^{2}=1-\left\{\frac{\sum_{i=1}^{m}{\left(y_{i}-\hat{y_{i}}\right)}^{2}}{\sum_{i=1}^{m}{\left(y_{i}-\bar{y_{i}}\right)}^{2}}\right\}$$

(21) $$MAPE=\frac{100\%}{N}\sum_{i=1}^{N}|\frac{y_{i}-\hat{y_{i}}}{y_{i}}|$$

where *m* is the length of the training and testing data for strain, $y_{i}$ denotes the measured true strain value, $\hat{y_{i}}$ represents the predicted strain value from the trained network, $\bar{y_{i}}$ is the average strain value, and N is the total number of prediction samples.

To address the mathematical limitations of the MAPE metric when applied to highly distorted or zero-crossing high-frequency strain data, two supplementary evaluation metrics are introduced: Normalized Root Mean Square Error (NRMSE) and Peak Error (PE). NRMSE effectively circumvents the instability of MAPE near zero values by normalizing the RMSE over the entire amplitude range. Furthermore, while this study focuses on advanced time-series forecasting rather than conducting downstream fatigue evaluations, capturing peak transient amplitudes accurately is essential to provide highly reliable data support for such future physical assessments. Therefore, PE is utilized to explicitly evaluate the model’s capability in preventing amplitude clipping under extreme vehicular impacts. These metrics are mathematically expressed as:

(22) $$NRMSE=\frac{RMSE}{y_{max}-y_{min}}$$

(23) $$PE=\frac{\left|max\left(y_{i}\right)-max\left({\hat{y}}_{i}\right)\right|}{max\left(y_{i}\right)}\times 100\%$$

where *y_max_* and *y_min_* denote the maximum and minimum values of the measured true strain sequence, respectively.

To quantify the reliability and statistical stability of the deterministic prediction results across the testing dataset, the Bootstrapping method was employed to compute the 95% Confidence Intervals (95% CIs) for all evaluation metrics. Specifically, for each model and prediction horizon, 1000 iterations of random resampling with replacement were executed on the paired sequences of actual and predicted strain values. In each iteration, the performance metrics were recalculated to generate an empirical distribution. Subsequently, the 2.5th and 97.5th percentiles of these distributions were extracted to establish the final 95% CIs. These intervals establish a mathematically rigorous bound on the expected model performance, definitively verifying the robustness of the proposed framework against potential sampling variability within the testing data.

## 3. Case Study

### 3.1. Engineering Background

**Figure 6:**
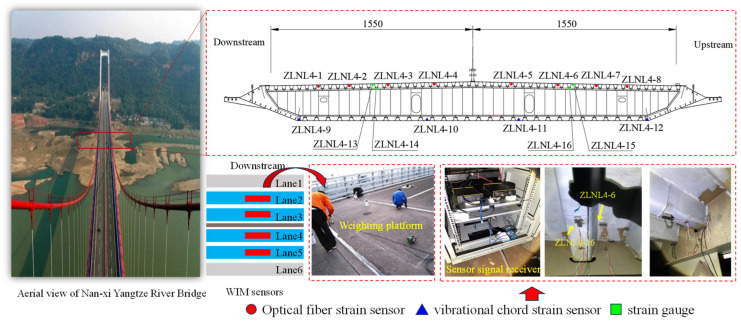
Sensor layout of Nan-xi Yangtze River Bridge.

The Nan-xi Yangtze River Bridge is a crucial control project of the Yibin–Luzhou Expressway in China. With a main span of 820 m, it boasts the longest main span of any bridge in Southwest China. The bridge’s main girder utilizes an orthotropic steel deck structure. A health monitoring system is installed on the Nan-xi Yangtze River Bridge, including a Weigh-in-Motion (WIM) system at the Luzhou tower location to record the arrival time, speed, and weight of vehicles on each of the four lanes. Fiber optic strain sensors are installed at critical weld details of the orthotropic steel deck: longitudinal stiffener-top plate welds, longitudinal stiffener-transverse diaphragm welds, and longitudinal stiffener-bottom plate butt welds. A total of 16 strain sensors (numbered ZLNL4-1 to ZLNL4-15) are deployed on the orthotropic steel deck, with a sampling frequency of 50 Hz. The specific arrangement of the bridge’s sensors is shown in Figure 6.

### 3.2. Strain Monitoring Data Preprocessing

**Figure 7:**
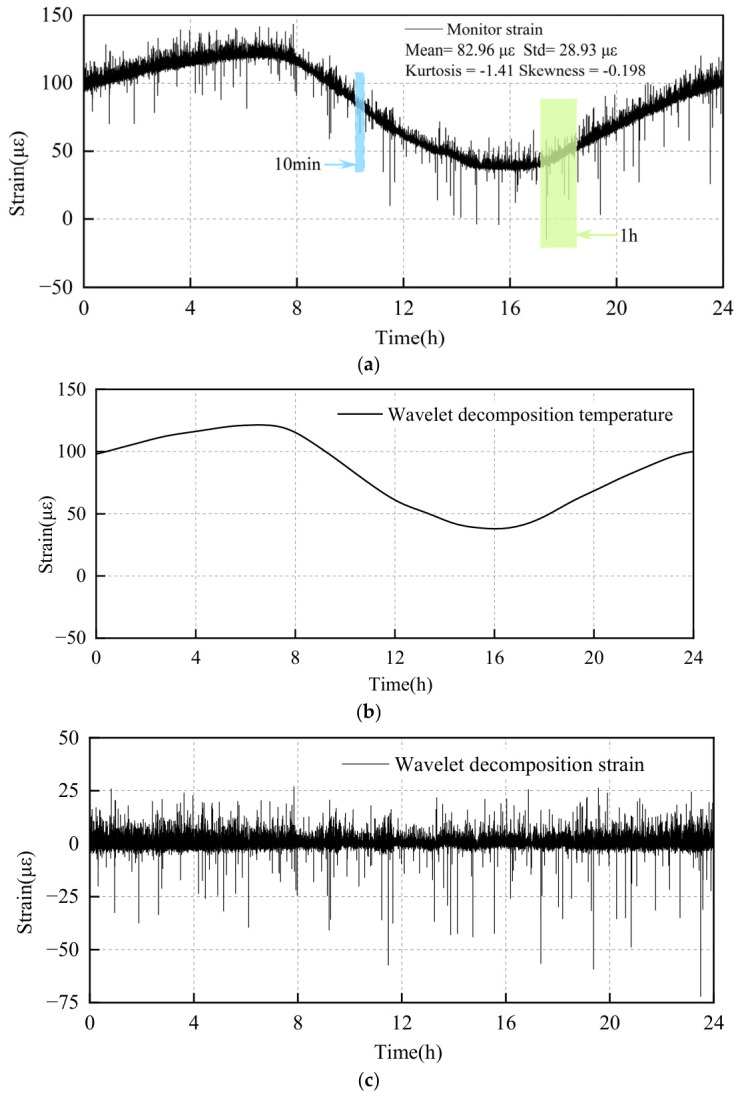

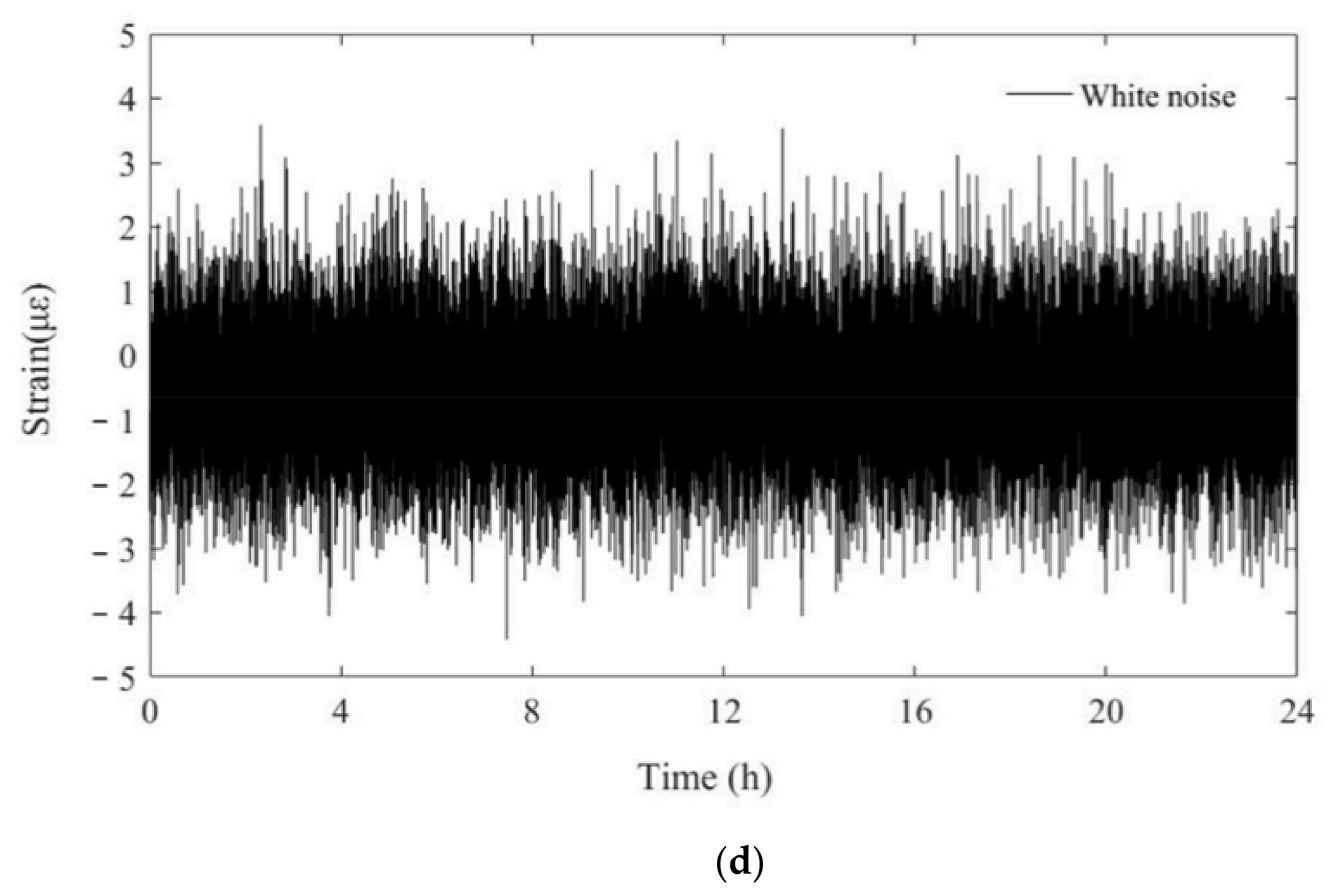
Separation of Strain Data from the ZLNL4-1 Using Wavelet Decomposition. (**a**) Initial monitoring strain time series; (**b**) Temperature-induced strain time series; (**c**) Vehicle loading-induced strain time series; (**d**) White noise-induced strain time series.

Figure 7 shows the raw monitored strain time series data from sensor ZLNL4-1 on 15 April 2024, along with the vehicle loading-induced strain, temperature-induced strain, and white noise strain time series after wavelet decomposition preprocessing. As shown in Figure 7a, the strain time series at the longitudinal stiffener-top plate weld of the orthotropic steel deck exhibits an overall sinusoidal pattern, completing one cycle within 24 h. This indicates that the strain data is primarily caused by environmental temperature effects. Figure 7b presents the temperature-induced strain time series data extracted from the monitored strain data. The larger, impulsive strain features observed in the raw monitored strain data are attributed to vehicle loads. Figure 7c shows the vehicle-induced strain after wavelet decomposition. The strain induced by white noise exhibits uniformly distributed, small, random strain data (See Figure 7d).

To isolate this structurally irrelevant white noise from the meaningful vehicle-induced high-frequency responses, a soft-thresholding filtration mechanism was applied to the detail coefficients during the wavelet reconstruction phase. Coefficients corresponding to these stochastic, low-amplitude measurement errors were zeroed out. Furthermore, this isolated white noise component is completely discarded prior to the prediction modeling. It is neither processed through a separate network track nor reintroduced into the final superimposed prediction, thereby ensuring that the deep learning architecture focuses exclusively on the deterministic physical behaviors of the bridge structure.

In the context of this supervised time-series forecasting framework, the ‘clean labels’ (ground truth targets) do not rely on manual annotations. Instead, they are defined as the continuous, physically meaningful strain components obtained under real-world operational conditions. Specifically, the raw data is acquired via fiber optic strain sensors on the Nan-xi Yangtze River Bridge operating at 50 Hz. The standard for data ‘cleanliness’ is mathematically guaranteed by the aforementioned 6-level wavelet decoupling and soft-thresholding process, which strictly isolates the deterministic thermal and vehicular responses from stochastic instrumental noise, thereby providing high-fidelity targets for model optimization.

**Figure 8:**
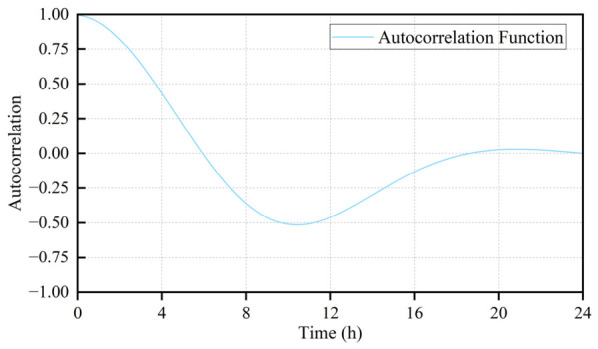
Autocorrelation plot of strain data.

To more deeply reveal the complex physical characteristics of the structural response of the orthotropic steel bridge deck, a comprehensive statistical analysis was conducted on the training dataset. The statistical results indicate that the mean of the strain sequence is 82.96 microstrain με, accompanied by a substantial standard deviation of 28.93 με, objectively reflecting the severe dynamic variations experienced by the bridge under coupled multi-source loads. Furthermore, the probability distribution of the strain data exhibits a slight negative skewness of −0.198 and a significantly negative kurtosis of −1.41. In the physical context of structural health monitoring, this typical platykurtic distribution demonstrates that the global variability of the strain signal is predominantly driven by slow-moving, large-amplitude, and low-frequency baseline drifts, namely the diurnal environmental temperature effects, rather than the discrete, sharp, transient strain peaks induced by random vehicle wheel loads. The Autocorrelation Function (ACF) plot of the strain data is presented in Figure 8. The ACF curve exhibits an extremely slow decay trend, maintaining high correlation coefficients throughout the entire cycle. This persistent, strong autocorrelation is a classic statistical hallmark of a strongly trend-dominated, highly non-stationary time series.

It is crucial to clarify the data resampling and synchronization strategy utilized in this study. While the raw strain data is physically acquired by the optical sensors at 50 Hz, directly feeding this massive volume into the deep learning architecture would cause severe computational redundancy. Therefore, the raw signals are downsampled to an effective rate of 1 Hz (yielding 86,400 points per 24 h, 3600 points per hour, and 600 points per 10 min). To prevent the loss of critical vehicle-induced transient peaks during this dimensionality reduction, a local block-maximum strategy is employed to preserve the maximum strain amplitude within each 1-s interval. Following this resampling, the aforementioned 6-level db10 wavelet transform acts as the core filtration mechanism to isolate structural responses from noise. Finally, the environmental temperature data and the discrete vehicle parameters from the WIM system are explicitly synchronized to this unified 1 Hz timeline via precise global timestamp alignment. For intervals without vehicle events, the dynamic WIM features are padded with zeros, thereby ensuring a continuous, dimensionally consistent multi-source input sequence.

### 3.3. Parameter Comparison of Deep Learning Models

To compare and validate the predictive performance of the models and better assess the advantages and disadvantages of the CNN-LSTM prediction model compared to other prediction models, this paper selects three representative time scales of strain data, 24 h, 1 h, and 10 min, for analysis.

To ensure the optimal performance of the proposed CNN-LSTM network while strictly preventing overfitting and implicit test-set tuning, a rigorous hyperparameter optimization procedure was employed utilizing the Hyperband algorithm. Hyperband dynamically allocates computational resources by initially evaluating a large configuration space over fewer epochs and successively halving the configurations based on their validation performance, thereby focusing exclusively on the most promising hyperparameter sets.

The search space for the algorithm was systematically defined across the network architecture. For the spatial feature extraction phase, the number of filters for the three Conv1D layers was searched from 64 to 256 in increments of 32, and the kernel sizes were alternated between 3 and 5. For the temporal sequence modeling phase, the LSTM units were searched between 50 and 100 in increments of 50. To introduce robust regularization, dropout rates for the LSTM layers were explored within the range of 0.2 to 0.5 in increments of 0.1. The learning rate of the Adam optimizer was logarithmically sampled between $10^{-6}$ and $10^{-2}$. Through this automated search, the optimal hyperparameters were determined, yielding a specific learning rate of 0.0085, along with 192 filters for the initial CNN layer and 50 units for the LSTM layers.

Crucially, to eliminate the risk of information leakage and implicit test-set tuning, the test data remained completely sequestered during the entire tuning and training phase. The hyperparameter selection was guided exclusively by the Mean Squared Error on an isolated validation set, which constituted a 20% hold-out from the training data. Furthermore, an early stopping mechanism was implemented. The training process continuously monitored the validation loss and was programmed to halt automatically if no improvement was observed over ten consecutive epochs. Upon triggering this early stopping protocol, the network restored the best-performing weights. To ensure a strictly unbiased comparative analysis, it is important to emphasize that this entire Hyperband hyperparameter optimization procedure, along with the early stopping mechanism and validation set isolation, was applied uniformly across all evaluated baseline models (CNN-GRU, LSTM, and GRU). This guarantees that all models reached their respective optimal capacities, ensuring the performance comparison is entirely fair and unbiased. This combination of an isolated validation set and early stopping ensures that the reported accuracy reflects the genuine generalization capability of the model on entirely unseen structural strain data.

Table 2 lists four neural network-based prediction models (CNN-LSTM, CNN-GRU, LSTM, and GRU) and their main hyper parameters. When constructing the optimal prediction model, Dropout, an effective regularization method, is widely used to mitigate overfitting in neural networks. During the model training phase, the dropout operation randomly sets a portion of the output units from the previous layer to zero with a certain probability, thereby reducing the complex co-adaptation relationships between neurons and enhancing the model’s generalization ability. However, during the inference or prediction phase, the dropout operation is not applied to maintain output stability and accuracy.

**Figure 9:**
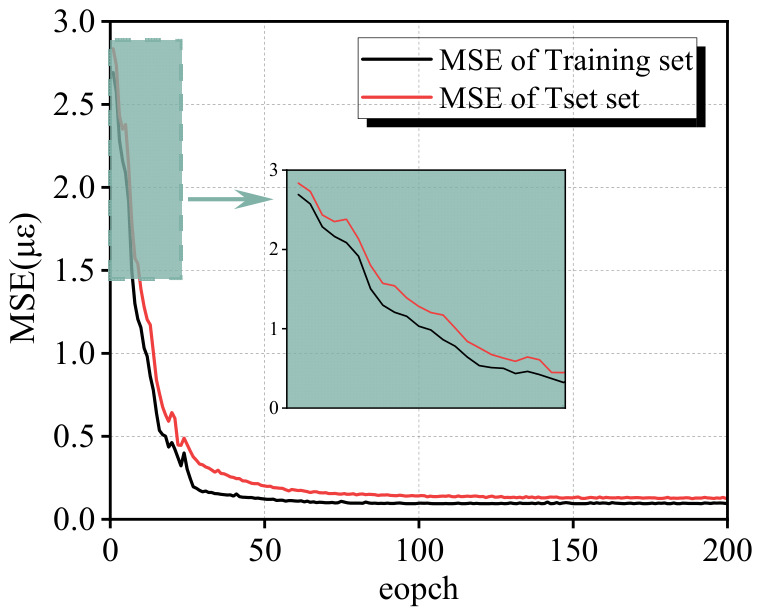
Loss curves of CNN-LSTM.

In addition to dropout regularization, a gradient decay strategy was employed to prevent the model from falling into local optima. Specifically, after the network structure training reached 100 epochs, the learning rate was adjusted to half its current value. This strategy helps the model converge more smoothly in the later stages of training. During this phase, other hyper parameters requiring optimization were set to moderate values. Subsequently, these hyper parameters were further optimized based on the best prediction results to determine the optimal hyper parameter combination, minimizing the prediction error on the validation set. Based on this optimal hyper parameter combination, the training and validation loss curves for the CNN-LSTM model during training are shown in Figure 9.

Multivariate linear regression analysis verified the good generalization performance among the input features of the dataset. When using the CNN-LSTM network, the time step *i* is a key hyper parameter affecting model performance. To determine the optimal time step, we substituted *i* from 1 to 30 into the four network models (CNN-LSTM, CNN-GRU, LSTM, and GRU) and evaluated the quality of the prediction results using the RMSE, MSE, and MAE.

**Figure 10:**
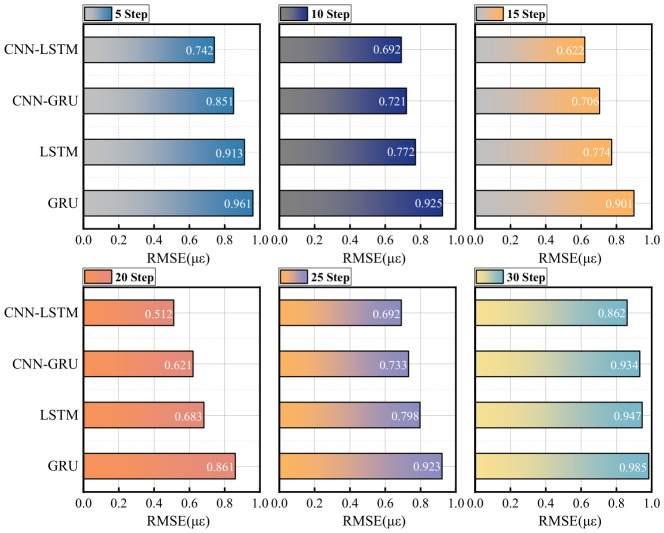
Relation between Input Steps and Output Error.

The length of the time series not only affects the model’s fitting performance but also determines the number of data points in the time dimension, thereby influencing the number of hidden layer units. In the multi-source data prediction experiment based on CNN-LSTM, comparative experiments with different time steps determined the optimal input length. When the time step i = 20, all four models achieved their best prediction performance, with CNN-LSTM exhibiting particularly superior results. Therefore, we set i = 20 as the optimal input time step for the model, as shown in Figure 10.

### 3.4. Correlation Analysis of Input Data Sets

**Figure 11:**
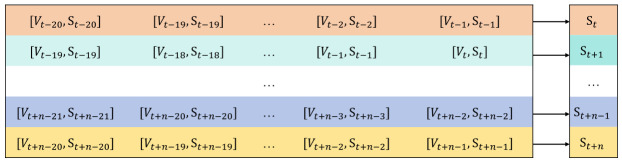
The relationship between network model input and output data.

Because the on-site monitored strain data is affected by environmental temperature, vehicle loads, and other uncertain factors, using only environmental temperature and vehicle load to construct a high-precision model is insufficient. Therefore, in this study, we incorporate vehicle load-related influencing factors for joint prediction analysis. Through in-depth study of the monitoring data, it was found that short-term strain sequences were closely related to vehicle effects, while long-term strain changes were mainly influenced by environmental temperature. Therefore, the monitoring data exhibits significant temporal correlation, and the time-varying relationship between the data is shown in Figure 11.

**Figure 12:**
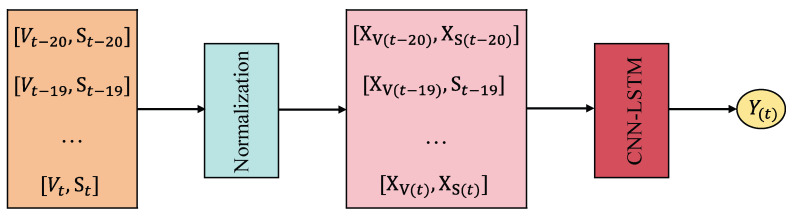
Data flow in CNN-LSTM network forward propagation.

Numerous factors contribute to the strain in the bridge main girder, and these factors are correlated with data points from the previous moment. Based on this observation, we use historical strain data to supplement the deficiencies in temperature-induced strain and vehicle effect information and mitigate the influence of various uncertain factors. Therefore, the neural network construction process becomes a dual-driven process of data and mechanism, enhancing the model’s explainability. To enhance the dual-driven capability of the framework, we integrate critical parameters from the Weigh-in-Motion (WIM) system: vehicle weight, speed, and axle count. These factors are normalized and concatenated with the processed strain sequences as multi-channel inputs. Specifically, for each time step in the input window, the CNN-LSTM architecture receives a fused vector consisting of the historical strain observations and the concurrent vehicle load parameters. This augmentation forces the CNN layers to map the latent relationship between external mechanical excitation and the internal structural response, effectively bridging the gap between purely data-driven time-series prediction and the underlying physics of bridge structural behavior. When using the CNN-LSTM network with a time step of 20 to model the strain caused by vehicle factors, we tested the effects of different input data lengths, different parameter features, and different hidden layer combinations. Through this process, we are able to directly apply the optimal hyper parameters to the current model. The data flow of forward propagation with the already optimized hyper parameters is shown in Figure 12.

### 3.5. Deep Learning Model Performance Comparison

**Figure 13:**
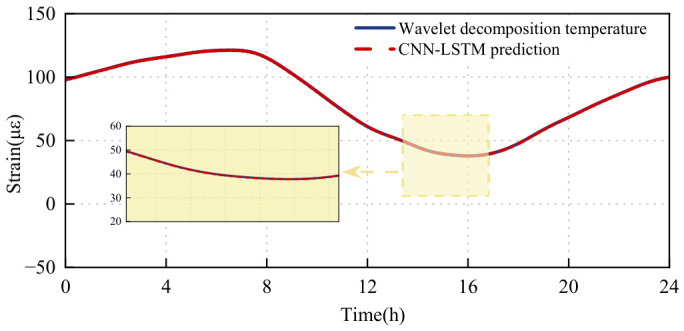
Comparison of measured and predicted temperature-induced strain data.

**Figure 14:**
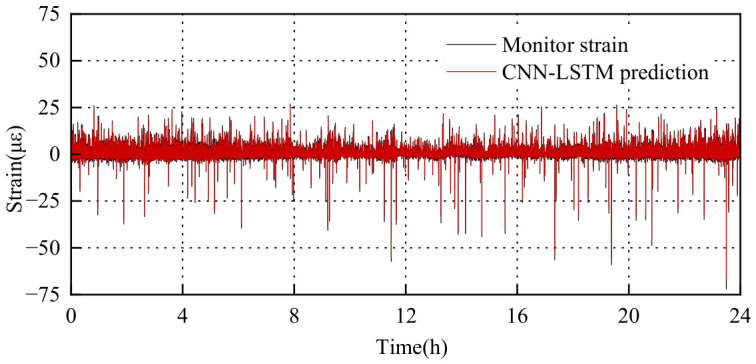
Comparison of measured and predicted vehicle loading-induced strain data.

To verify the generalization ability of the CNN-LSTM model framework, this study focuses on examining its prediction performance at different time scales. We selected the 24-h data from Figure 7a as a sample, totaling 86,400 data points. To strictly prevent data leakage and look-ahead bias in time-series forecasting, the continuous structural strain dataset is partitioned chronologically into three completely isolated subsets: a training set, a validation set, and a test set. Specifically, the initial 70% of the continuous sequence is strictly designated as the training set, which is exclusively utilized to optimize the trainable weights of the CNN-LSTM architecture. The subsequent 10% of the data serves as the validation set. This subset is employed solely to monitor the convergence behavior and trigger the early stopping mechanism, thereby preventing overfitting without exposing the model to future information. Finally, the remaining 20% of the dataset is reserved as the test set. This final subset is completely sequestered during the entire training and hyperparameter tuning phases, being utilized only once for the final, unbiased assessment of the predictive framework’s generalization capability. This strict chronological isolation ensures that the evaluation metrics mathematically reflect the true predictive accuracy of the model under entirely unseen operational conditions. Figure 13 shows the prediction results of the CNN-LSTM model for temperature-induced strain, demonstrating its ability to effectively predict the overall trend of the data. Figure 14 shows the prediction results of the CNN-LSTM model for vehicle-induced strain, similarly showing effective prediction of the overall trend. After separately predicting the temperature-induced strain and vehicle-induced strain, we superimpose the two prediction results to obtain a complete prediction result for the real-time monitoring data.

Before evaluating the predictive performance, it is necessary to clarify the rationale behind selecting CNN-GRU, LSTM, and GRU as the primary comparative baselines. While the literature review covers a broad spectrum of methodologies, traditional algorithms (e.g., ARIMA, SVR) were excluded from this benchmark due to their well-documented limitations in capturing highly non-linear, high-frequency transient peaks in massive SHM datasets. Conversely, highly complex architectures like Transformers or GANs, although powerful, introduce substantial computational overhead and parameter burdens, which fundamentally contradict the real-time, lightweight edge-deployment objective of this proposed framework. Therefore, LSTM, GRU, and CNN-GRU were strategically selected to serve as direct structural ablation baselines. This rigorous selection explicitly isolates and validates the specific performance gains contributed by the 1D-CNN feature extractor and the distinct memory gating mechanisms within the proposed hybrid architecture.

To explicitly evaluate the structural contribution and necessity of the wavelet decomposition step within the proposed cascaded architecture, an ablation study was conducted. Specifically, the performance of the proposed comprehensive framework was compared against an identical CNN-LSTM network trained directly on the raw, undecomposed structural strain data. Table 3 presents the quantitative ablation results for the 24-h prediction task. As evidenced by the ablation metrics, eliminating the wavelet decomposition step leads to a severe degradation in predictive accuracy. For instance, the MAPE surges significantly from 0.4134% to 0.6177%, the RMSE escalates from 0.512 to 0.726, and the *R*^2^ drops abruptly from 0.961 to 0.914. This substantial performance gap highlights the inherent difficulty of learning directly from raw structural strain signals. In operating bridge decks, the raw strain is a deeply coupled mixture of high-frequency, sharp transient peaks and low-frequency, large-amplitude drifts. The transient peaks are induced by random vehicle axle loads, whereas the baseline drifts are driven by environmental thermal variations. Forcing a neural network to simultaneously map these two physically conflicting phenomena from a single sequence deeply complicates the optimization landscape, leading to feature confusion and higher residual errors. By integrating the db10 wavelet transform as a physical-prior decoupling mechanism prior to the deep learning module, the complex non-stationary signal is explicitly separated into its deterministic thermal baseline and stochastic vehicular components. This targeted decomposition significantly simplifies the representational learning task for the subsequent CNN-LSTM layers, allowing the spatial convolutions to focus purely on local vehicular gradients and the LSTM to accurately track independent temporal trends. Consequently, the ablation study statistically and physically verifies that the wavelet decomposition is an indispensable structural pillar of the proposed high-fidelity prediction framework.

**Figure 15:**
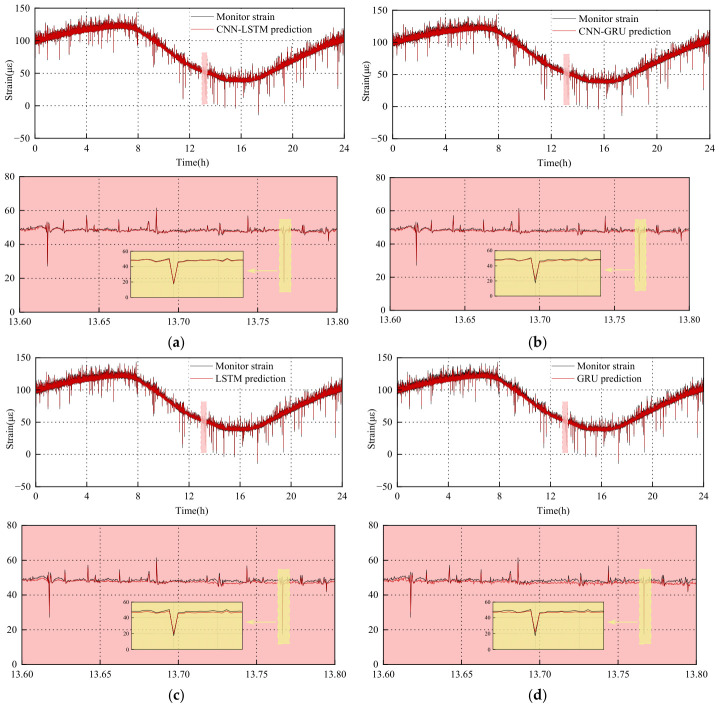
Comparison of measured and predicted strain data using 4 types of deep learning models. (**a**) CNN-LSTM; (**b**) CNN-GRU; (**c**) LSTM; (**d**) GRU.

**Figure 16:**
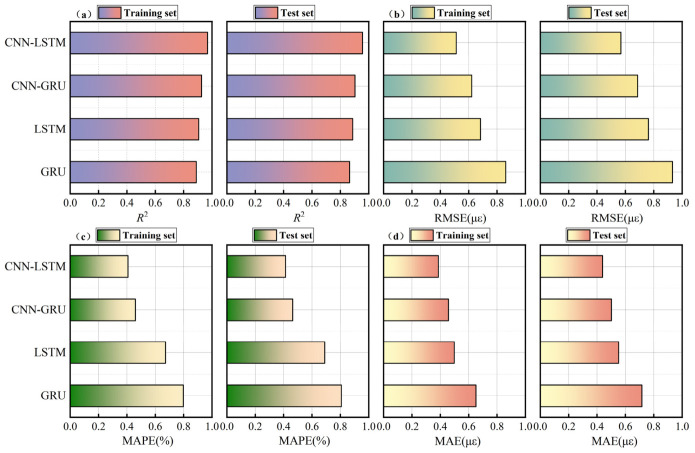
Comparison of four types of model evaluation indexes on a 24 h time scale. (**a**) Bar comparison of the coefficient of determination (*R*^2^) for the training set and test set; (**b**) Bar comparison of the coefficient of determination (*R*^2^) for the training set and test set; (**c**) MAPE (in percentage) of all candidate models across training and test partitions; (**d**) MAE (unit: με) yielded by the four models on the training set and test set.

Figure 15 shows a comparison curve of the 24-h monitored strain data and the predicted time series from the CNN-LSTM, CNN-GRU, LSTM, and GRU models. Figure 16 shows that the CNN-LSTM model’s strain time series prediction has the highest fitting degree compared to the other three deep learning models. Especially near the extreme points of the strain data, the CNN-LSTM model demonstrates a stronger ability to capture strain extremes. Furthermore, the CNN-LSTM model can effectively quantify prediction uncertainty and provide relatively accurate prediction results, comprehensively reflecting the data trend. The prediction effect of the CNN-GRU model is second only to the CNN-LSTM model, while the GRU model has the largest overall prediction error.

As shown in Figure 16, these performance indicators evaluate the generalization ability of the CNN-LSTM model. Under large-scale data prediction, the RMSE, MSE, and MAE values of CNN-LSTM are smaller, indicating higher prediction accuracy. Figure 16 shows that the *R*^2^ value is close to 1, indicating a good fit between the predicted data recovered by the model and the actual measured data. Specifically, for the amount of data in continuous prediction, the RMSE increments of CNN-GRU, LSTM, and GRU compared to CNN-LSTM are 21.3%, 33.3%, and 68.2%, respectively; the MSE increments are 47.3%, 78.2%, and 182.8%, respectively; and the MAE increments are 40%, 77.3%, and 83.5%, respectively. Table 4 shows that as the time scale increases, the correlation between data sequences gradually decreases, leading to a decrease in network prediction accuracy. The comparison results show that the proposed CNN-LSTM algorithm has the best interpolation performance among all methods, with an *R*^2^ of 0.961, the highest among the four models, indicating that this network model has the best prediction accuracy.

The computed 95% CIs further substantiate the superiority of the proposed cascaded architecture for long-term forecasting. As detailed in the statistical performance results for the 24-h prediction horizon, the CNN-LSTM framework consistently yields the most compact confidence bounds among all evaluated candidates. Specifically, the proposed model achieves a MAPE of 0.4134% with a 95% confidence interval ranging from 0.4083% to 0.4379%. This margin is significantly lower and tighter than the corresponding interval observed for the standard LSTM baseline, which ranges from 0.6695% to 0.7076%. Furthermore, the RMSE of the CNN-LSTM is maintained within a narrow interval of 0.497 to 0.533, whereas the LSTM baseline exhibits a broader and higher interval between 0.675 and 0.694.

Overall, the prediction results show that the CNN-LSTM method exhibits strong effectiveness and generalization ability when handling multi-source data prediction, further verifying the reliability and superiority of this method.

**Figure 17:**
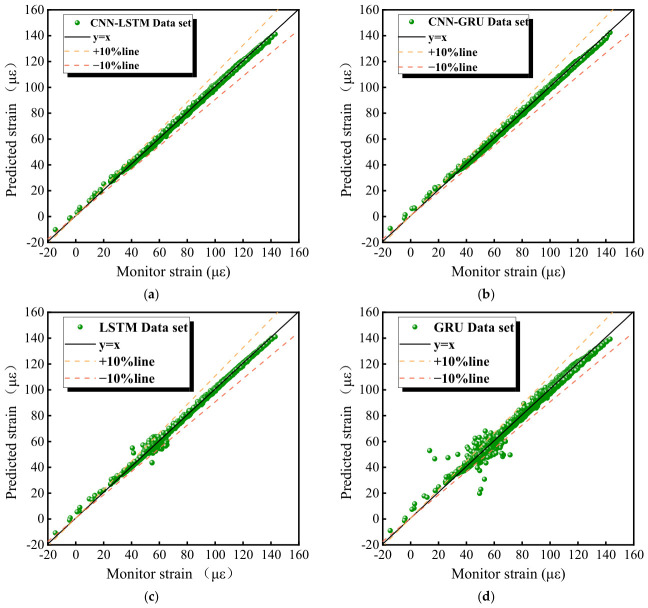
Linear fitting diagram of 10% error interval on 24 h time scale. (**a**) CNN-LSTM; (**b**) CNN-GRU; (**c**) LSTM; (**d**) GRU.

Considering the storage, transmission, and computational burden of long-term bridge monitoring data, we discussed the data input processing method in the above examples. First, by separating the temperature effects from the measured strain data, we distinguished the dynamic and static responses caused by vehicles. It is noteworthy that the proposed method is also applicable to larger datasets, but this requires a longer training time and more computational resources. To more intuitively compare the performance of different models in strain data prediction, Figure 17 presents the linear fitting results of the CNN-LSTM, CNN-GRU, LSTM, and GRU models under the same data scenario. Each subplot shows the scatter plot distribution of measured and predicted values, along with a reference line (*y* = *x*) and ±10% error lines.

From Figure 17, it can be observed that the predicted values of the four models generally show high correlation with the measured values, with the scatter points uniformly distributed on both sides of the linear regression curve. In contrast, the CNN-LSTM model performs best, with most of its data points clustered closely around the *y* = *x* line and almost entirely within the ±10% error range, indicating high prediction accuracy. The CNN-GRU model is second best; the fitting degree of its predicted values is slightly inferior to that of CNN-LSTM, and some data points are more dispersed, but still mainly concentrated within the error range. The fitting effect of the LSTM model further decreases, and the scatter points deviate more significantly from the reference line, with a small number of data points exceeding the ±10% error line. The GRU model has the worst prediction accuracy; its data points are more dispersed, and the fitting degree between the predicted and measured values is the lowest, with the most points deviating from the error range. The results show that the CNN-LSTM model, when processing bridge strain data prediction, can learn more accurate underlying features, and its prediction performance is significantly superior to other models. This further verifies the effectiveness and advantages of CNN-LSTM in complex time-series tasks.

**Figure 18:**
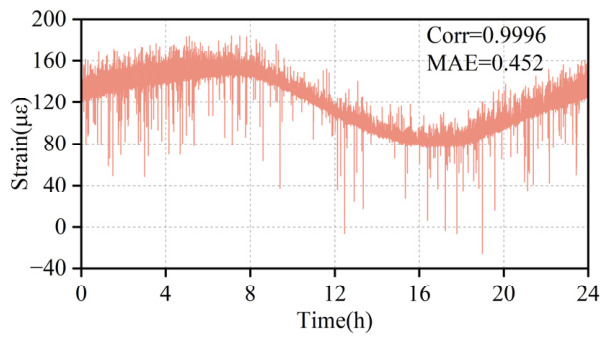
The 24-h strain history curve of the ZLNL4-7 strain sensor.

To demonstrate the general applicability and robustness of the proposed physics-guided cascaded framework, it is essential to evaluate its performance across diverse environmental conditions and structural locations. Regarding environmental and operational robustness, the framework has been rigorously validated across a continuous 24-h testing horizon. This 24-h cycle inherently encompasses significant diurnal temperature gradients and dramatic shifts in traffic densities, such as the stark contrast between daytime peak heavy-truck loads and nighttime sparse traffic. The consistently narrow confidence intervals and low prediction errors, highlighted by a 24-h MAPE of 0.4133%, statistically confirm that the model is highly resilient to shifting daily environmental and operational conditions. Furthermore, to validate the spatial robustness of the methodology, an additional evaluation was conducted using strain data acquired from a structurally distinct sensor, designated as ZLNL4-7, located on the same orthotropic steel bridge deck. The sensor strain data is shown in Figure 18. Different monitoring points inherently exhibit unique localized stiffness, boundary conditions, and traffic load influence lines. Consequently, validating the framework on a new sensor rigorously tests its capability to generalize beyond a single specific structural detail. The proposed CNN-LSTM methodology was directly applied to predict the strain responses at this new location. The evaluation results demonstrate that the framework successfully maintained its high-fidelity predictive performance, achieving an impressive $R^{2}$ of 0.955. This high level of accuracy is highly consistent with the exceptional performance observed at the primary sensor location. This additional validation mathematically confirms that the proposed feature decoupling and sequence modeling methodology is not overfitted to the specific time-series characteristics of a single monitoring point. Instead, the framework demonstrates strong spatial generalizability, proving its suitability for widespread deployment across various critical fatigue evaluation details within the bridge structure.

## 4. Results and Discussion

### 4.1. Strain Data Prediction in 1-h Time Window

To further verify the generalization ability of the CNN-LSTM model framework, this section explores its prediction performance at 1-h and 10-min time scales. Taking the one-hour data from Figure 7a as an example, a total of 3600 data points were used. Consistent with the overall methodology, the data was partitioned chronologically, with the first 70% strictly designated as the training set, the subsequent 10% serving as the validation set, and the remaining 20% reserved as the test set to verify the prediction accuracy of the trained model.

**Figure 19:**
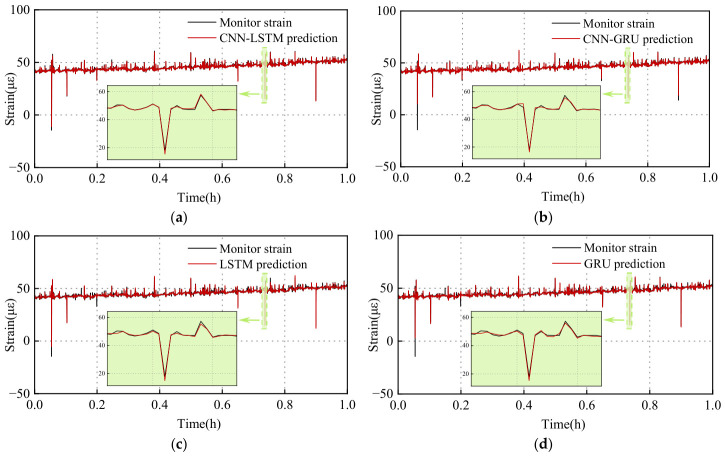
Comparison of four types of predicted curves on a 1 h time scale. (**a**) CNN-LSTM; (**b**) CNN-GRU; (**c**) LSTM; (**d**) GRU.

Figure 19 shows the results of the four models, CNN-LSTM, CNN-GRU, LSTM, and GRU, in short-term strain prediction. Comparative analysis shows that within a time window with relatively small strain fluctuations, there are certain errors between the predicted values and the actual monitored values for all four models. However, the prediction performance of the CNN-LSTM framework is significantly superior to the other three models. Specifically, the predicted values are very close to the measured values, indicating that the CNN-LSTM method exhibits outstanding stability and prediction accuracy. Especially at data extremes, the CNN-LSTM model can better capture strain extremes. At the same time, this model also performs excellently in handling uncertainty and can predict the data trend relatively completely.

**Figure 20:**
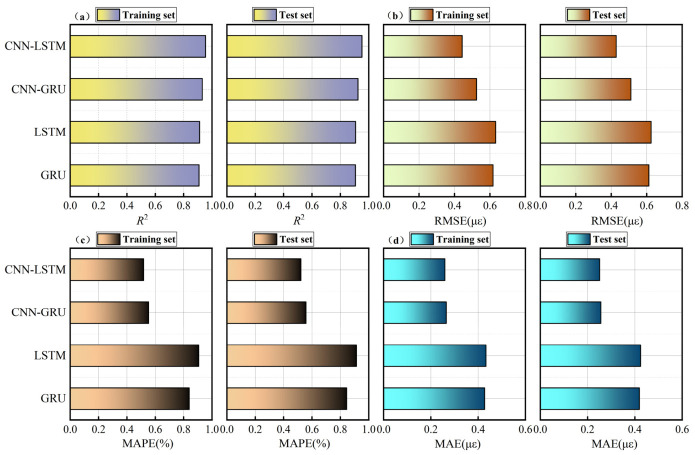
Comparison of four types of model evaluation indexes on a 1 h time scale. (**a**) R^2^ distributions of CNN-LSTM, CNN-GRU, LSTM and GRU on training and test sets; (**b**) Comparative results of RMSE (με) from the above four models in training and testing stages; (**c**) MAPE (%) comparisons of different models between training data and test data; (**d**) MAE (με) contrast of the four selected models for training and test samples.

Figure 20 shows that in continuous prediction data, the RMSE increments of the CNN-GRU, LSTM, and GRU models relative to the CNN-LSTM model are 18.3%, 42.7%, and 39.3%, respectively; the MSE increments are 40.3%, 103.6%, and 94.4%, respectively; and the MAE increments are 2.3%, 67.2%, and 64.9%, respectively. The data in Table 5 shows that as the time scale increases, the correlation between data sequences gradually decreases, leading to a decrease in prediction accuracy. The comparison results show that the proposed CNN-LSTM algorithm exhibits the best interpolation performance among all methods, with an *R*^2^ of 0.952, ranking first among the four models, indicating that this network model has a significant advantage in accuracy. This indicates that the CNN-LSTM method has strong effectiveness and strong generalization ability when handling multi-source data.

### 4.2. Strain Data Prediction in 10 m Time Window

**Figure 21:**
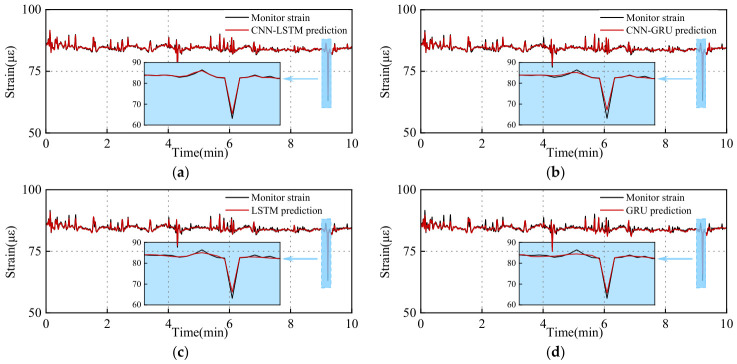
Comparison of four types of predicted curves on a 10 m time scale. (**a**) CNN-LSTM; (**b**) CNN-GRU; (**c**) LSTM; (**d**) GRU.

Using the 10-min time-scale data from Figure 7a, comprising 600 data points, predictions were made. Following the identical tripartite chronological split (70% for training, 10% for validation, and 20% for testing), Figure 21 shows the specific prediction results of the four models, CNN-LSTM, CNN-GRU, LSTM, and GRU, in short-term strain prediction. From a visual comparative analysis, it can be seen that within periods of relatively small strain fluctuations, there is a certain degree of error between the prediction results of the four models and the actual monitored values. However, the CNN-LSTM framework significantly outperforms the other three models, exhibiting better prediction performance. Comparing the predicted and measured values in Figure 21, it is found that the prediction results of CNN-LSTM almost coincide with the original measured data, indicating that this method has high stability and accuracy. Especially in the extreme regions of the strain data, the CNN-LSTM model exhibits a stronger ability to capture extreme values. At the same time, this model can effectively quantify the uncertainty in the prediction and accurately predict the overall trend of the data.

**Figure 22:**
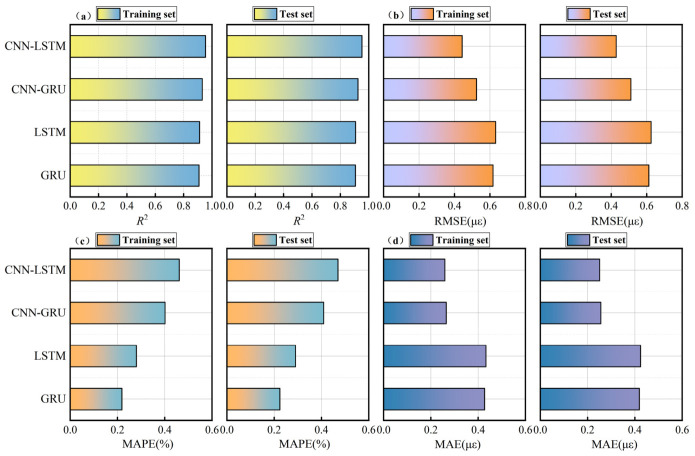
Comparison of four types of model evaluation indexes on a 10 m time scale. (**a**) Bar comparison of R2 values reflecting the fitting performance of CNN-LSTM, CNN-GRU, LSTM and GRU; (**b**) Cross-model comparison of RMSE (unit: με) on training data and test data; (**c**) Distribution of MAPE (%) to quantify the relative prediction deviation of each model; (**d**) MAE (unit: με) comparison that displays the absolute prediction error of different models.

Figure 22 shows that in continuous prediction scenarios, the RMSE increments of the CNN-GRU, LSTM, and GRU models compared to CNN-LSTM are 23.9%, 50.2%, and 76.1%, respectively; the MSE increments are 53.3%, 125.2%, and 210.3%, respectively; and the MAE increments are 19.3%, 51.7%, and 73.1%, respectively. Table 6 shows that as the time scale increases, the correlation between data sequences weakens, leading to a decrease in the model’s prediction accuracy. By comparing the performance of each model, the proposed CNN-LSTM model exhibits the best interpolation performance, with an *R*^2^ of 0.946, superior to the other three models, indicating that CNN-LSTM performs best in prediction accuracy. Overall, the CNN-LSTM method exhibits strong generalization ability in multi-source data prediction tasks and performs excellently.

While the proposed CNN-LSTM framework demonstrated lower prediction errors across metrics such as the MAPE, RMSE, and MAE compared to the baseline sequence models, it is imperative to verify that these performance improvements are statistically significant rather than artifacts of random testing-set fluctuations. To rigorously validate this, a two-step statistical testing procedure was performed on the absolute prediction errors across the 10-min, 1-h, and 24-h prediction horizons. Initially, a one-way repeated-measures analysis of variance was conducted to evaluate the global performance differences among the four evaluated models. Across all evaluated horizons, the test yielded *p*-values far below the 0.001 threshold, indicating a highly significant divergence in overall model predictive capacity. Subsequently, post-hoc paired comparisons were executed using one-sided paired *t*-tests to specifically assess the error reduction achieved by the cascaded models versus the standard recurrent networks. The results indicate that the CNN-LSTM significantly outperformed the standard LSTM baseline, with all associated *p*-values being consistently lower than $10^{-10}$ across all time scales. Furthermore, in both the 10-min and 24-h predictions, the CNN-LSTM framework statistically emerged as the best-performing architecture among all candidates with a confidence level exceeding 99.9%. This comprehensive multi-scale statistical validation mathematically confirms that the proposed integration provides a fundamental, statistically robust enhancement in structural monitoring accuracy that is entirely independent of random sample variations.

### 4.3. Computational Efficiency and Real-Time Deployability

A fundamental prerequisite for deploying data-driven models in continuous structural health monitoring is that the framework must satisfy strict computational and real-time processing constraints. To quantitatively substantiate the practical deployability of the proposed method, a comprehensive evaluation of model complexity, offline training time, and online inference speed was conducted across the evaluated architectures. All benchmarks were executed in a standardized computational environment featuring an NVIDIA RTX 4060 Ti graphics processing unit and 32 gigabytes of random-access memory.

As shown in Table 7, the baseline pure recurrent networks exhibit relatively low structural complexity. The proposed CNN-LSTM framework introduces additional convolutional layers for spatial feature decoupling, thereby increasing the total parameter count to approximately 360,109 (0.36 M). However, within the context of edge-deployable algorithms, this footprint of approximately 0.36 M parameters is exceedingly lightweight, demanding negligible memory overhead for field-site hardware integration. Notably, despite possessing the largest parameter volume among all evaluated architectures, the CNN-LSTM framework maintains a highly satisfactory training efficiency, requiring a mere 42 s per epoch for offline optimization. This remarkable computational efficiency is primarily attributed to the highly parallelizable nature of the spatial convolution modules, which significantly compress the temporal sequence length before feeding the features into the LSTM, effectively offsetting the computational burden introduced by the added parameters. Consequently, this highly efficient offline optimization phase poses absolutely no limitation to the practical engineering deployment of the model.

Crucially, the runtime performance of the framework strictly satisfies all constraints of real-time monitoring. The measured online inference latency for the CNN-LSTM model is approximately 0.63 milliseconds per sequence sample. Considering the typical physical acquisition intervals of sensors in practical engineering (which generally range from 10 to 20 milliseconds), this inference latency is more than an order of magnitude shorter than the data generation interval, thereby completely eliminating the risk of processing backlogs. This quantitative comparison rigorously corroborates that the proposed cascaded architecture successfully achieves an optimal equilibrium between exceptionally high predictive accuracy and the strict computational efficiency mandated by uninterrupted, real-time structural state tracking.

Furthermore, the practical engineering value and generalization capability of the proposed Wavelet-CNN-LSTM framework are highlighted in several aspects for real-world Structural Health Monitoring (SHM) applications. First, by explicitly separating temperature-induced variations from transient vehicle impacts via physics-guided wavelet decoupling, the model possesses high environmental and operational adaptability, allowing it to be generalized to bridges in different climatic zones or with varying traffic volumes without altering the network core. Second, as evidenced by the low parameter count and high computational efficiency in Table 7, the framework can be easily deployed onto resource-constrained edge computing gate-ways for real-time, on-site strain forecasting. Third, due to the structural universality of the 1D-CNN feature extractors, this approach can be horizontally extended to predict the strain time series of other key fatigue-prone components in orthotropic steel bridge decks, such as longitudinal ribs and diaphragm cutouts, demonstrating strong cross-component engineering scalability.

## 5. Conclusions

This study presents a physics-guided cascaded deep learning framework integrating db10 wavelet decoupling with a CNN-LSTM architecture for high-fidelity strain time series prediction in orthotropic steel bridge decks. Based on comprehensive experimental validation using real-world monitoring data from the Nan-xi Yangtze River Bridge, the primary conclusions are drawn as follows:

(1) By leveraging the high vanishing moments of the db10 wavelet transform, the highly non-stationary raw strain is explicitly separated into a deterministic low-frequency thermal baseline and stochastic zero-mean vehicular transient components. This framework effectively resolves the feature extraction conflicts and interferences inherent in traditional black-box end-to-end models when processing conflicting physical characteristics.
(2) Across distinct time scales of 10 min, 1 h, and 24 h, the proposed CNN-LSTM architecture consistently demonstrates superior predictive performance compared to baseline models, including CNN-GRU, LSTM, and GRU. The MAPE is strictly maintained below 0.6% across all tested horizons, with an R^2^ reaching 0.961, confirming exceptional reliability.
(3) Despite its deep cascaded nature, the CNN-LSTM module remains exceedingly lightweight with approximately 0.36 million parameters. It achieves an offline optimization speed of 42 s per epoch and a single-step inference latency of merely 0.63 milliseconds. The framework mathematically ensures zero processing backlogs, strictly satisfying the rigorous computational constraints required for continuous real-time bridge monitoring.
(4) The proposed framework exhibits strong generalization capability and outstanding advantages for practical engineering scenarios. It balances low computational latency with high physical interpretability, making it highly suitable for deployment in edge-computing-based bridge health monitoring systems and scalable to other similar steel structures.

Similar modifications have been applied to accurately reflect that the proposed framework serves as a fundamental prerequisite for downstream fatigue analysis, rather than a standalone fatigue evaluation tool.

Despite the research progress described above, the conclusions of this paper are still limited by the scope of the current experimental validation. Specifically, regarding its usage boundaries, the proposed framework has currently been evaluated exclusively at sensor locations on steel bridge decks under normal operational conditions. A primary limitation is its dependency on the training data distribution; it cannot be directly extrapolated to out-of-distribution extreme load events (e.g., severe sudden structural damage or extreme weather anomalies) that are absent from the initial training dataset. To overcome this inherent limitation of data-driven models, future research will focus on exploring how to achieve low-cost, rapid deployment of this framework across diverse bridge structures. The goal is to transform the current pre-trained framework into a highly generalizable foundational model capable of achieving high-precision predictions for new bridges with vastly different boundary conditions and spatial characteristics, requiring only a minimal amount of target-domain data for fine-tuning.

Despite the promising predictive performance demonstrated in this study, several limitations must be acknowledged. First, the current empirical validation relies primarily on continuous monitoring data spanning a limited timeframe (e.g., 24 h) from specific sensing nodes. While this dataset adequately validates the core algorithm’s capability to capture diurnal thermal cycles and random traffic impacts, it lacks comprehensive multi-condition validation across diverse seasonal environments, widespread sensor locations, and extreme mechanical loading scenarios. Future research will focus on establishing an extensive, cross-seasonal, and cross-bridge monitoring dataset to further evaluate and fortify the model’s spatiotemporal generalization robustness and structural reliability under extreme operational conditions.

## Figures and Tables

**Table 1:** Comparison of Strengths and Weaknesses of Time Series Prediction Algorithms.

Typical Models	Key Advantages	Critical Limitations
ARIMA	Mathematically interpretable	Poor at modeling highly nonlinear data
SVR	Fast computation	Cannot handle non-stationary dynamic data
ANN	Suitable for small datasets	Lacks temporal memory mechanism
RNN	Excellent at learning nonlinearities	Suffers from vanishing gradients
LSTM	Solves vanishing gradients	Sluggish in capturing sudden, high-frequency vehicle strain peaks
GRU		
GAN	Powerful for data augmentation	High computational cost and training instability
GNN	Captures complex spatial topology	Computationally excessive for single-point forecasting
Transformer	Strong global context representation	Treats physical signals and noise equally without physical decoupling
Wavelet-CNN-LSTM	Captures transient peaks (CNN) and long-term trends (LSTM)	Requires careful empirical tuning for wavelet basis and decomposition levels

**Table 2:** Parameters of Neural Networks.

Deep Learning Model	Hyper Parameters	Values
CNN-LSTM	Number of neurons in convolution layer 1	128
	Number of neurons in convolution layer 2	192
	Number of neurons in convolution layer 3	128
	Number of neurons in the LSTM 1 layer	50
	Dropout1	0.3
	Number of neurons in the LSTM 2 layer	100
	Dropout2	0.2
	Learning rate	0.0085
CNN-GRU	Number of neurons in convolution layer 1	128
	Number of neurons in convolution layer 2	192
	Number of neurons in convolution layer 3	128
	Number of neurons in the GRU 1 layer	50
	Dropout1	0.3
	Number of neurons in the GRU 2 layer	100
	Dropout2	0.2
	Learning rate	0.0085
LSTM	Number of neurons in the LSTM 1 layer	50
	Dropout1	0.3
	Number of neurons in the LSTM 2 layer	100
	Dropout2	0.2
	Learning rate	0.0085
GRU	Number of neurons in the GRU 1 layer	50
	Dropout1	0.3
	Number of neurons in the GRU 2 layer	100
	Dropout2	0.2
	Learning rate	0.0085

Note: ‘Conv’ denotes the 1D convolution layer.

**Table 3:** The 24-h prediction performance comparison with and without wavelet decomposition.

Deep Learning Model	RMSE	MAPE	MAE	$R^{2}$
Wavelet + CNN-LSTM	0.512	0.4134%	0.439	0.961
CNN-LSTM	0.726	0.6177%	0.683	0.914

**Table 4:** Model evaluation index parameters of 24 h predicted data.

Deep Learning Model	RMSE [95%CI]	MAPE(%) [95%CI]	MAE [95%CI]	$R^{2}$	NRMSE	PE (%)
CNN-LSTM	0.512 [0.497, 0.533]	0.4134% [0.4083, 0.4379]	0.439 [0.428, 0.451]	0.961	0.0068	1.39%
CNN-GRU	0.621 [0.613, 0.632]	0.4637% [0.4483, 0.4942]	0.511 [0.501, 0.523]	0.922	0.0082	2.38%
LSTM	0.683 [0.675, 0.694]	0.6896% [0.6695, 0.7076]	0.748 [0.741, 0.756]	0.897	0.0091	2.41%
GRU	0.861 [0.848, 0.872]	0.8067% [0.7916, 0.8254]	0.779 [0.767, 0.792]	0.884	0.0114	1.43%

**Table 5:** Model evaluation index parameters of 1 h predicted data.

Deep Learning Model	RMSE	MAPE (%)	MAE	$R^{2}$	NRMSE	PE (%)
CNN-LSTM	0.443	0.5214%	0.259	0.952	0.0156	0.002%
CNN-GRU	0.524	0.5573%	0.265	0.924	0.0184	0.77%
LSTM	0.632	0.9132%	0.433	0.907	0.0222	0.69%
GRU	0.617	0.8441%	0.427	0.906	0.0217	2.08%

**Table 6:** Model evaluation index parameters of 10 m predicted data.

Deep Learning Model	RMSE	MAPE (%)	MAE	$R^{2}$	NRMSE
CNN-LSTM	0.327	0.2241%	0.238	0.946	0.0115
CNN-GRU	0.405	0.2902%	0.284	0.919	0.0142
LSTM	0.491	0.4093%	0.361	0.893	0.0173
GRU	0.576	0.4699%	0.412	0.876	0.0202

**Table 7:** Comparison of model complexity and computational efficiency.

Model	Total Parameters	Inference Speed (ms/Sample)	Relative Training Time (per Epoch)
CNN-LSTM	360,109 (0.36 M)	0.63 ms	42 s
CNN-GRU	290,909 (0.29 M)	0.55 ms	37 s
LSTM	71,301 (0.07 M)	0.39 ms	29 s
GRU	53,951 (0.05 M)	0.41 ms	24 s

## Data Availability

Some or all data, models, or code that support the findings of this study are available from the corresponding author upon reasonable request.
